# The chromosome-level genome assembly of the giant dobsonfly *Acanthacorydalis orientalis* (McLachlan, 1899)

**DOI:** 10.1038/s41597-024-03194-3

**Published:** 2024-04-08

**Authors:** Mingming Zou, Aili Lin, Yuyu Wang, Ding Yang, Xingyue Liu

**Affiliations:** 1 Department of Entomology, China Agricultural University, Beijing, 100193 China; 2 College of Plant Protection, Hebei Agricultural University, Baoding, 071001 China

**Keywords:** Genome evolution, Phylogenetics

## Abstract

*Acanthacorydalis orientalis* (McLachlan, 1899) (Megaloptera: Corydalidae) is an important freshwater-benthic invertebrate species that serves as an indicator for water-quality biomonitoring and is valuable for conservation from East Asia. Here, a high-quality reference genome for *A. orientalis* was constructed using Oxford Nanopore sequencing and High throughput Chromosome Conformation Capture (Hi-C) technology. The final genome size is 547.98 Mb, with the N50 values of contig and scaffold being 7.77 Mb and 50.53 Mb, respectively. The longest contig and scaffold are 20.57 Mb and 62.26 Mb in length, respectively. There are 99.75% contigs anchored onto 13 pseudo-chromosomes. Benchmarking Universal Single-Copy Orthologs (BUSCO) analysis showed that the completeness of the genome assembly is 99.01%. There are 10,977 protein-coding genes identified, of which 84.00% are functionally annotated. The genome contains 44.86% repeat sequences. This high-quality genome provides substantial data for future studies on population genetics, aquatic adaptation, and evolution of Megaloptera and other related insect groups.

## Background & Summary

Aquatic insects, which host at least 9.5% of animal species on Earth^1^, are of great interest for ecological and evolutionary studies due to their notable adaptation to the freshwater habitats and pivotal functions in the freshwater ecosystem. Studies of aquatic insects in a genomic perspective can provide a fundamental basis for understanding their evolutionary history and adaptive mechanisms in the freshwater ecosystem.

**Fig. 1:**
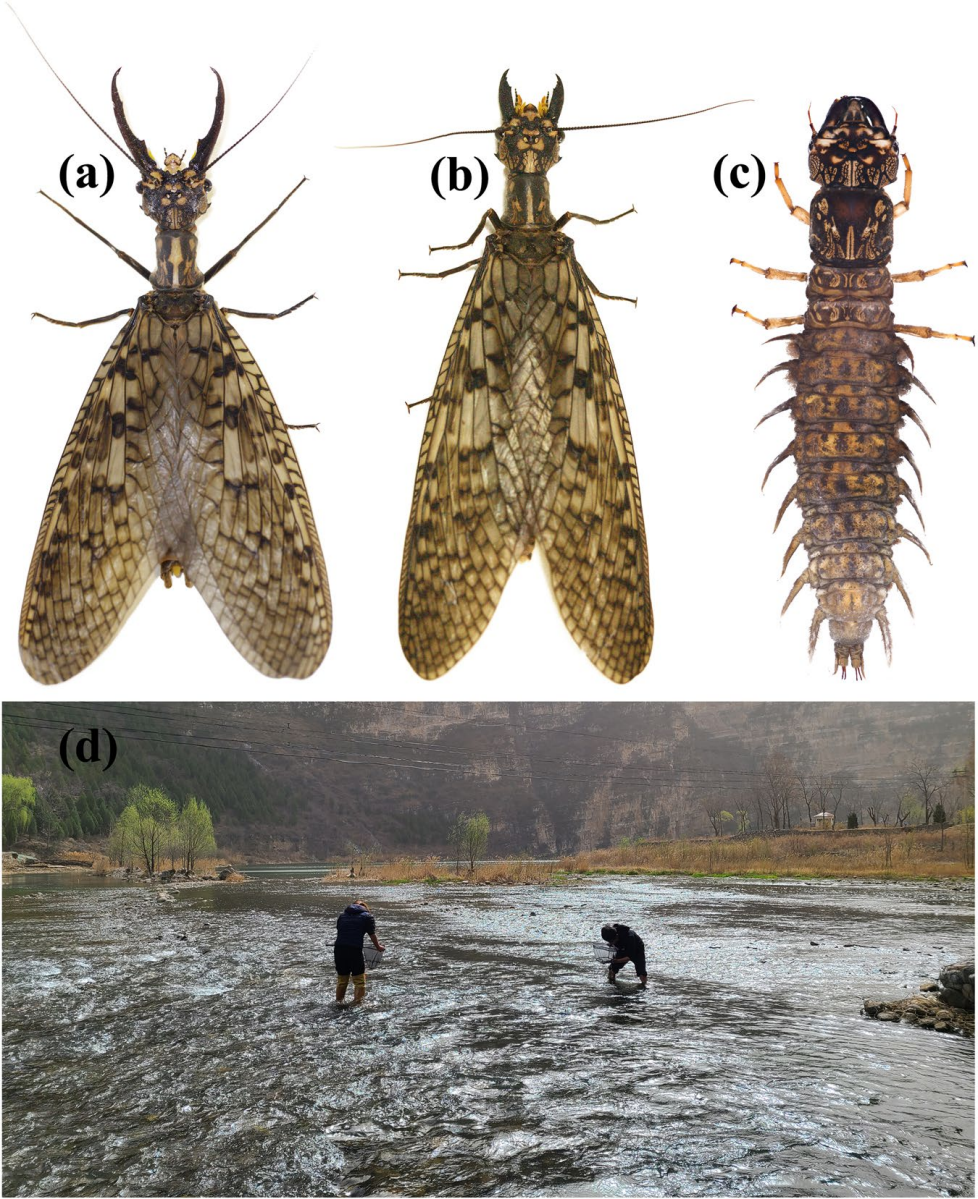
Habitus and habitat photos of *Acanthacorydalis orientalis*. (**a**) Male adult. (**b**) Female adult. (**c**) Larva. (**d**) Collecting site of sequenced specimen. (Photos were taken by Weiwei Zhang, Yuezheng Tu, and Xingyue Liu).

Megaloptera (dobsonflies, fishflies and alderflies) belongs to the superorder Neuropterida and is one of the archaic groups of Holometabola. Currently, there are 34 genera and approximately 400 extant species worldwide, sorted in two families^2^. According to fossil records, both extant families of Megaloptera (Sialidae and Corydalidae) originated at least from the Upper Triassic^3^. Larvae of Megaloptera are exclusively aquatic and inhabit various clean freshwater habitats as benthic predators, which are important bioindicators of freshwater quality^4,5^. Some species of Corydalidae are spectacular due to the huge body-size, with wingspan around 200 mm^6–8^. The East Asian endemic genus *Acanthacorydalis* van der Weele, 1907 is among such large-sized corydalids, with remarkable sexual dimorphism in adult mandibles^9^ (Fig. 1). *Acanthacorydalis orientalis* (McLachlan, 1899) is the most widespread species in this genus and ranges from Southwestern and Central China to Northern China. The larvae of *A. orientalis* inhabit larger rocky rivers with fast running water^10^ (Fig. 1). In Southwestern China, *A. orientalis* larvae and other large-sized corydalid larvae are used as food and medicine for local people^11^, while currently facing overhunting. In terms of the peculiar morphological features and threatened situation, *A. orientalis* has been listed as protected animal species in some areas (e.g., Beijing) of China.

So far, the whole genomic data of aquatic insects mainly refer to specific species of Odonata, Ephemeroptera, Trichoptera, and aquatic Coleoptera and Diptera, such as damselflies^12^, mayflies^13^, caddisflies^14^, aquatic fireflies^15^ and chironomids^16^. Currently, only one chromosome-level genome with a size of 480.67 Mb of the Asian dobsonfly species *Neoneuromus ignobilis* has been reported^17^. Based on this genome, convergent expansions of blue-sensitive and long wavelength-sensitive opsins, sulfotransferases, as well as thermal stress response TRP channels in aquatic insects have been found through comparative genomic analysis. Moreover, evidence of molecular convergences in aquatic insects during convergent amino acid substitutions and gene family evolution was also provided. For the aquatic chironomid larvae which can tolerant water pollution, expansion of the gene family related to detoxification metabolism has been found with adaptation to such hazard environment. Conversely, as the corydalid larvae are sensitive to the deterioration of freshwater habitat, uncovering the gene family related to detoxification metabolism of corydalid species is crucial for understanding the specific preference for clean freshwater habitats of Megaloptera.

Here, we assembled a high-quality chromosome-level genome of *A. orientalis* by using Oxford Nanopore sequencing and High throughput Chromosome Conformation Capture (Hi-C) technology. The final genome size is 547.98 Mb, with the N50 value of contig being 7.77 Mb. Benchmarking Universal Single-Copy Orthologs (BUSCO) analysis showed that the completeness of the genome assembly is 99.01%. There are 10,977 protein-coding genes identified. BUSCO analysis showed that the completeness of the genome annotation is 95.76%. The assembly and annotation of this genome demonstrated a high degree of continuity and integrity. The genome herein reported will shed light on the aquatic adaptation of the megalopteran larvae as well as other benthic invertebrates and the genetics and evolution of this archaic holometabolan group.

## Methods

### Sampling and sequencing

The larva of *A. orientalis* used in this study was collected in Juma River, Shisandu, Fangshan District, Beijing, China on March 28, 2021. The genomic DNA was extracted by the G2 method using QIAGEN® Genomic Kit (Cat#13343, QIAGEN). The DNA was purified after extraction due to impurities.

For Nanopore sequencing, 1D library was constructed with LSK109 kit on a PromethION sequencer. There are 153.75 Gb data processed (coverage: 280.57 X) totally. For Circular Consensus Sequencing (CCS), SMRTbell library was constructed with SMRTbellTM Express Template Prep Kit 2.0. Long DNA fragments of the SMRTbell library were sequenced on a PacBio Sequel II sequencer and the insert fragment size was 15 kb. The HiFi (High fidelity) reads were obtained by using CCS version 4.2.0 (--min-passes 1 --min-rq 0.99 --min-length 100) to process the offline data of PacBio Sequel II sequencer. One SMRT cell was processed resulting 416.42 Gb of subreads (coverage: 759.91 X). There are 25.36 Gb of HiFi reads (mean length: 14.66 kb, N50 length: 14.68 kb) obtained from the PacBio Sequel II platform for genome assembly polishing after calling CCS. For next-generation sequencing (NGS), the library was constructed by using the MGIEasy DNA kit, with an insert fragment size of 300–500 bp. The DNA library was sequenced using 150 bp paired-end (PE) reads on the MGI-2000 platform according to the protocol. There are 176.05 Gb (coverage: 319.44 X) raw data generated by the MGI 2000 platform.

The Hi-C technology was used to assist the genome assembly at the chromosome-level, which has been applied to capture whole genome chromatin interactions^18^. The Hi-C fragment library was constructed and sequenced using the Illumina Nova-Seq 6000 platform according to the previously published protocol^19^. Hi-C analysis was performed using tissues from the same larva. The Hi-C library was sequenced with PE reads of 150 bp (insert fragment size was 300–500 bp). There are 53.04 Gb of Hi-C raw data (176,791,896 PE reads) generated totally.

For RNA-seq, the RNA library was constructed with Qiagen Kit using 500 ng RNA with 12.02 Gb raw data (coverage: 21.93 X) obtained from the MGI-2000 platform. For PacBio Iso-seq sequencing, 500 ng RNA was reverse transcribed into cDNA and amplified using Iso-seq Express Oligo Kit with cDNAs purified using ProNex Beads, and a library was constructed using BluePippin with an insert fragment size of 0.5–6 kb. There are 55 Gb raw data (coverage: 100.36 X) obtained.

### Genome size estimation and assembly

The raw data of NGS reads which sequenced on the MGI-2000 platform were filtered by using fastp version 0.21.0^20^ (n 0-f 5-f 5-t 5-t 5-q 20) preprocessor to remove low-quality reads and obtain clean data. The quality of clean data was controlled using FastQC version 0.11.8^21^ (--extract). Then a part of the clean data after quality control was used for genome survey and all clean data was used to correct the genome assembly.

The genome survey analysis was conducted to infer the genomic characteristics of *A. orientalis* and develop a reasonable assembly plan before assembling the genome. There are 112.46 Gb MGI DNA raw data used for k-mer analysis to estimate the genome size and heterozygosity. The frequency distribution analysis on quality filtered reads was performed using KMC version 3.1.1^22^ (- k35 ci1-cs1000000) with 35-mer. The short segment data at corresponding depths was simulated using the genome of Arabidopsis. The heterozygosity of *A. orientalis* was estimated by performing k-mer curve fitting under different gradient combinations of heterozygosity. The genome size is approximately 431.01 Mb and the heterozygosity is 1.2% based on the frequency distribution analysis of 35-mer (Supplementary Table 1).

On the Nanopore sequencing platform, the process of converting potential signals generated by DNA or RNA strands passing through nanopores into corresponding base sequences is called base-calling. The fastq format of raw reads with mean_qscore <7^23^ was filtered using the official tool Guppy version 3.2.2 + 9fe0a78^24^ (-c dna_r9.4.1_450bps_fast.cfg) to obtain the pass reads. Then the pass reads can be directly used for subsequent assembly. The genome was assembled using the NextGraph (default parameter) module in NextDenovo version 2.3.1 (default parameters, reads_cutoff as 1k,seed_cutoff as 50k) (https://github.com/Nextomics/NextDenovo) after correcting and trimming the raw data using NextCorrect (default parameter) module. A genome draft of 567.31 Mb was generated through denovo assembly. The genome preliminary assembly was corrected using Nextpolish version 1.3.0^25^ with default parameter. The Nanopore third-generation data were corrected three times and the PacBio HiFi reads were corrected three times, while NGS data were corrected four times. The polished genome size is 551.29 Mb and contig N50 is 7.82 Mb after decontamination.

High-quality reads were obtained by filtering the original off-line data of the sample. The reads mapped uniquely to the genome at both ends of PE for subsequent analysis were extracted after removing the duplicate reads. The effective interaction and the proportion of sequences with self-cycle and biotin at the end was predicted using the position information of the DpnII site in the genome sketch. Finally, the genome assembly was assisted by analyzing the interaction between sequences. The data obtained by sequencing was the raw off-line sequence containing sequencing connector sequence and low-quality sequence. Fastp version 0.21.0^20^ (- n 0-f 5-f 5-t 5-t 5-q 20) was used to filter the original sequence to ensure the quality of the analytic data and obtain high-quality clean reads. Then the duplicate reads were removed before the subsequent analysis. There are 53.04 Gb of Hi-C raw data (176,791,896 PE reads) generated after filtering and 52.46 Gb (coverage: 95.73 X) of Hi-C filtered data (350,924,920 clean PE reads) used to assist the chromosome-level assembly.

**Fig. 2:**
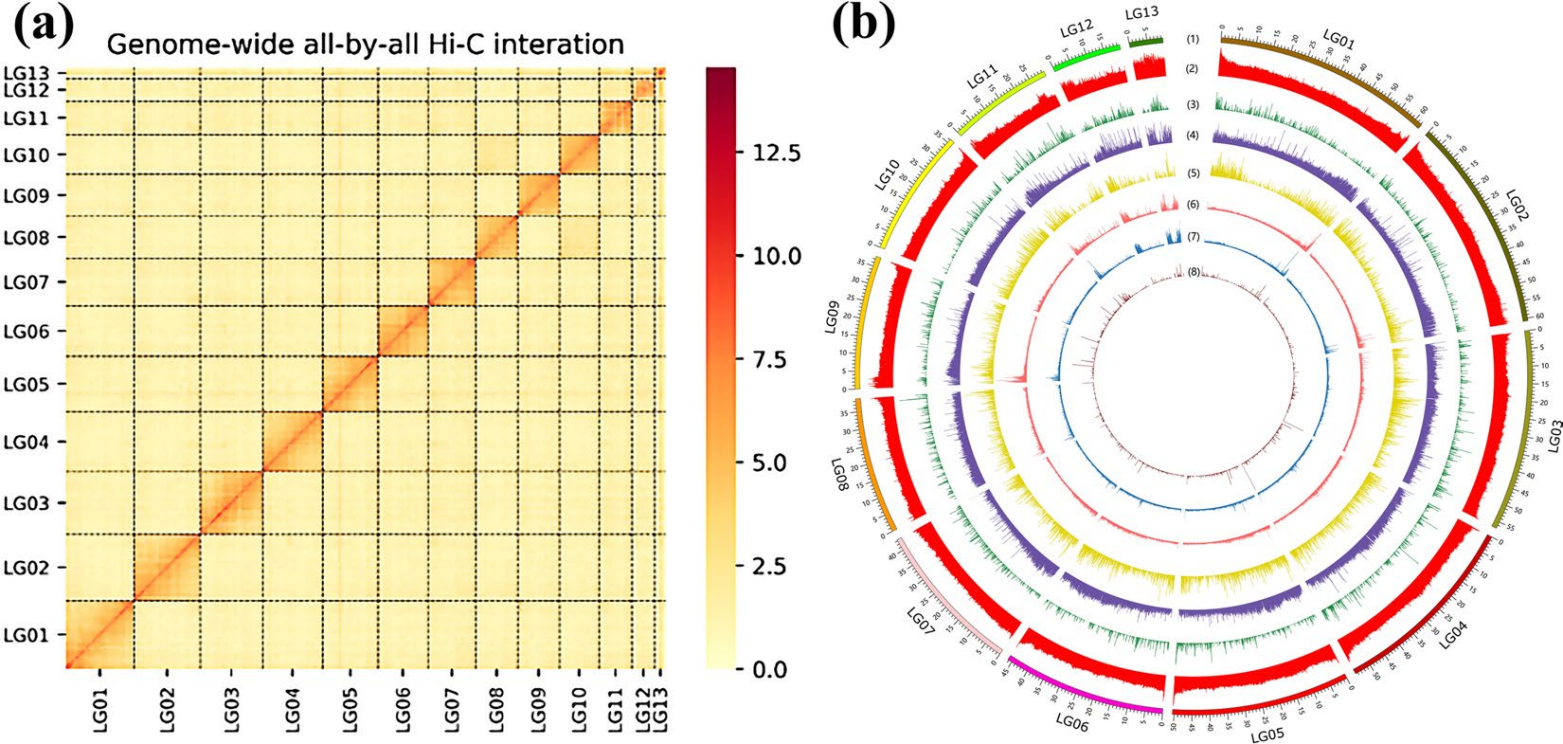
Hi-C contact map and overview of the genomic landscape of *Acanthacorydalis orientalis*. (**a**) The heatmap shows the strength of interval interactions within pseudo-chromosomes. Resolution: 100 kb. The frequency of Hi-C interactive links is represented by color, ranging from yellow (low) to black (high). (**b**) Distribution of genomic features of *A. orientalis*. Blocks on the outmost circle represent all 13 pseudo-chromosomes of *A. orientalis*. Peak plots from outer to inner circles represent the length of each pseudo-chromosome (1), the GC content of each pseudo-chromosome (2), protein coding genes (3), the density of repeat sequences (DNA elements (4); SINE, short interspersed elements (5); LINE, long interspersed elements (6); LTR, long terminal repeat elements (7); simple repeats (8)), respectively.

Single-ended alignment of the sequenced Reads1 and Reads2 with the assembled genome sequence was performed to obtain localization information using Bowtie2 version 2.3.2^26^ (alignment mode: - end to end; parameter: --very sensitive - L 30) due to the unique nature of the construction of the Hi-C library. Then the linked sites (enzyme cleavage point reconnection) in the unmapped PE reads after comparison were found by interception and comparison again. Finally, the PE reads of two comparisons were merged and the proportion of uniquely mapped PE reads was calculated. The unique read pairs around the DpnII cleavage site for comparison were determined by comparing and analyzing them. Hi-C interaction signals were used as a measure of the degree of correlation between different contigs by standardizing the DpnII cleavage sites on the genome sketch. For the genome sketch with a karyotype of 2n, using LACHESIS^27^ (https://github.com/shendurelab/LACHESIS) software, the contig sequence of the sketch was clustered into 13 pseudo-chromosomal groups using an agglomerative hierarchical clustering algorithm. Contig sorting was performed within the cluster group of each pseudo-chromosome. Finally, the final chromosome-level genome sequence was obtained by adding 100 N connections after sequence and direction of the contig determined. There are 100,691,193 unique PE reads retained, including 65,757,980 effective interaction PE reads after mapping them onto the genome draft. There are 170 contigs attached to 13 pseudo-chromosomes (Fig. 2a). The length of 13 pseudo-chromosomes ranged from 9.51 Mb to 62.25 Mb (Fig. 2b), respectively, and the total length of 13 pseudo-chromosomes is 546,614,392 bp, accounting for 99.75% of the genome size (Table 1). The size of the chromosome-level genome obtained ultimately is 547.98 Mb, with 184 contigs and 27 scaffolds. The longest contig and scaffold is 20.56 Mb and 62.25 Mb, respectively, and the N50 length of the contig and scaffold is 7.77 Mb and 50.53 Mb, respectively.

**Table 1:** Pseudo-chromosomes length in the genome of *Acanthacorydalis orientalis*.

Pseudo-chromosomes ID	Size (bp)	Number of scaffolds
LG01	62,255,071	16
LG02	60,753,524	13
LG03	56,959,169	16
LG04	54,725,841	12
LG05	50,529,619	10
LG06	46,286,187	9
LG07	43,316,481	7
LG08	38,688,734	6
LG09	37,858,193	9
LG10	36,286,860	8
LG11	29,682,556	19
LG12	19,762,091	25
LG13	9,510,066	20
Total	546,614,392	170

Contigs were sorted within the cluster group of each pseudo-chromosome. In the final chromosome-level genome obtained, 100 kb was taken as a bin. The number of Hi-C read pairs covering any two bins was used as the intensity signal for the interaction between two bins. In the Hi-C interaction heatmap of pseudo-chromosomes, the heatmap coordinates represented pseudo-chromosomes, and the color of each point represented the log value of the corresponding genome bin pair interaction intensity, which increased sequentially from yellow to black.

### Gene structure and functional annotation

SSR sequences in the genome were analyzed using GMATA version 2.2^28^ with default parameters since it could identify microsatellite sites in sequences. Tandem repeats (TR) in the genome were analyzed using TRF version 4.07b^29^ with default parameters. The Trans-posable elements (TE) of this species were predicted using RepeatMask version 1.331^30^ (http://www.repeatmasker.org) based on the final-constructed repeat sequence database. There are 120,522 TRs with a total length of 4,359,538 bp (Supplementary Table 2), accounting for 0.80% of the whole genome. The total length of 2,401,248 TEs is 231,229,263 bp, accounting for 42.20% of the whole genome. The four most abundant classes of TEs include DNA elements (29.63%), long interspersed elements (LINEs) (5.10%), miniature inverted-repeat transposable elements (MITEs) (4.32%) and long terminal repeats (LTRs) (2.19%) (Fig. 2b,Supplementary Table 2). There are 2,601,889 repeats in total, accounting for 44.86% of the whole genome, with the length of 245.85 Mb after integrating TRs, TEs and other repeats.

Gene structure prediction mainly used homologous protein prediction, transcriptome prediction, and ab initio prediction. The corresponding protein information with the genome was compared, and the predicted results of all homologous species were integrated using GeMoMa version 1.6.1^31^ with default parameters based on the protein sequence information of related species to obtain the structural information of the corresponding predicted genes. The proteins of related species, i.e., *Tribolium castaneum* (Coleoptera)^32^, *Coccinella septempunctata* (Coleoptera)^33^, *Abscondita terminalis* (Coleoptera)^34^, *Harmonia axyridis* (Coleoptera)^35^, *Cryptolaemus montrouzieri* (Coleoptera)^36^, *Nebria riversi* (Coleoptera)^37^, *Polypedilum vanderplanki* (Diptera)^38^, *Stenopsyche tienmushanensis* (Trichoptera)^14^, *Chrysopa pallens* (Neuroptera)^39^ and *Chrysoperla carnea* (Neuroptera)^40^, were downloaded from GenBank for homology-based gene prediction (Table 2). There are a total of 16,875 genes predicted based on the results (Table 3).

**Table 2:** Information of 11 species used in this study.

	Species	NCBI accession	Size/ Number of proteincoding genes
Coleoptera	*Tribolium castaneum*^32^	GCA_000002335.3	165.9 Mb / 14,322
Coleoptera	*Coccinella septempunctata*^33^	GCA_907165205.1	398.8 Mb / 16,932
Coleoptera	*Harmonia axyridis*^35^	GCF_914767665.1	425.5 Mb / 18,548
Coleoptera	*Abscondita terminalis*^34^	GCA_013368085.1	499.7 Mb / 20,439
Coleoptera	*Cryptolaemus montrouzieri*^36^	GCA_013387265.1	988.1 Mb / 27,858
Coleoptera	*Nebria riversi*^37^	GCA_018344505.1	147.4 Mb / 17,895
Diptera	*Polypedilum vanderplanki*^38^	GCA_018290095.1	119 Mb / 17,863
Trichoptera	*Stenopsyche tienmushanensis*^14^	GCA_008973525.1	451.5 Mb / 14,672
Neuroptera	*Chrysopa pallens*^39^	GCA_020423425.1	538.4 Mb / 12,840
Neuroptera	*Chrysoperla carnea*^40^	GCA_905475395.1	560.2 Mb / 15,864
Megaloptera	*Acanthacorydalis orientalis*	this study	547.98 Mb / 10,977

**Table 3:** Gene prediction results based on three strategies.

Prediction strategies	Software used	Total number of genes	Average gene length (bp)	Average CDS length (bp)	Average exons number per gene	Average exon length (bp)	Average intron length (bp)
De novo	AUGUSTUS	11,167	26,801.36	1,706.46	6.46	264.04	4,593.79
Homology	GeMoMa	16,875	23,398.98	1,265.94	4.59	275.79	6,164.91
RNA/Iso-seq	PASA	7,949	19,347.40	2,608.78	7.07	369.19	2,759.28
Final set	EVM	10,977	25,723.76	1,644.25	6.28	261.7	4,557.88

For RNA-seq-based gene prediction, the clean data was compared to the reference genome using STAR version 2.7.3a^41^ after data quality control. Then the transcripts of the clean data were assembled using StringTie version 1.3.4d^42^ with default parameters based on the results of the comparison genome and 6,702 second-generation transcript sequences were obtained for subsequent analysis. For ISO-seq-based gene prediction, high-quality reads of insert were obtained after the connector sequence was removed from PacBio offline data and the same polymerase read was self-corrected. The full-length transcript was identified and the primer sequence was removed using Lima version 2.2.0 (https://lima.how/) with default parameters. The full-length non-chimeric reads with the end poly-A sequence removed (full-length non-concatemer reads) were obtained by calling the Refine process in Isoseq 3 (https://github.com/ylipacbio/IsoSeq3). A cluster was performed on the full-length non-chimeric reads obtained after the refining. The high-quality consensus reads were mapped to the reference genome by using Minimap2 version 1.0^43^, and the sequences of multiple gene loci on the alignment were removed from the alignment. Then the subprogram in cDNA_Cupcake (https://github.com/Magdoll/cDNA_Cupcake/wiki) was used to filter and further remove the redundancy, and the alignment results before and after redundancy removal were counted. A total of 7,405 third-generation transcripts were obtained for downstream gene prediction and analysis. There are a total of 7,949 genes predicted (Table 3) using PASA version 2.3.3^44^ based on the second and third-generation transcripts obtained from the above analysis, and corresponding training models were obtained for ab initio prediction.

The species prediction model was obtained by selecting reliable genes for model training through AUGUSTUS version 3.3.1^45^ based on transcriptome prediction. There are 11,167 genes finally predicted (Table 3) using AUGUSTUS version 3.3.1^45^ for ab initio prediction of gene structure based on this training model.

The gene set of the initial genome of *A. orientalis* was obtained by integrating GeMoMa^31^ gene prediction results, PASA^44^ gene prediction results and ab initio gene prediction results, using Evidence Modeler (EVM) version 1.1.1^46^ (--segmentSize 1000000 --overlapSize 100000) with a certain weight value (EVM weights: PASA 10, GeMoMa 5, AUGUSTUS 1). The final gene set was obtained by removing genes that contain TEs and encoding errors from the initial genome gene set through TransposoonPSI (http://transposonpsi.sourceforge.net/). A total of 10,977 genes (Table 3) were predicted, with an average gene length of 25,723.76 bp and an average Coding DNA Sequence (CDS) length of 1,644.25 bp, while the average exons number per gene is 6.28 bp, the mean exon length is 261.7 bp and the mean intron length is 4,557.88 bp (Table 3), respectively.

Genomic ncRNA was predicted using Infernal version 1.1.2^47^ with default parameters compared with the Rfam database^48^, while tRNA was predicted using tRNAscan-SE version 2.0^49^ (--thread 4 -E -I) and rRNA and its various subunits were predicted using RNAmmer version 1.2^50^ (-S euk -m lsu,ssu,tsu -gff) to construct the model based on the assembled genome sequence. The above results were further integrated to obtain the predicted ncRNA in the genome. There are 1,153 ncRNA sequences annotated in total, including 156 rRNAs, 190 small RNAs, 15 cis-regulatory elements, and 792 tRNAs (Supplementary Table 3).

Gene functional annotation was completed by comparing with public databases including SwissProt^51^, NR, KEGG^52,53^, KOG^54^, and Gene Ontology (GO)^55^. There are 9,221 genes (84.00%) annotated functionally (Table 4).

**Table 4:** Statistics for functional annotation of protein-coding genes.

Database	Number	Percent (%)
Swiss-Prot	8,225	74.93
KEGG	6,027	54.91
KOG	7,043	64.16
GO	6,146	55.99
NR	9,053	82.47
Total	9,221	84

## Data Records

The raw Nanopore, PacBio, Hi-C, MGI, RNA-seq, and Iso-seq data were submitted to the Sequence Read Archive at NCBI under accession numbers SRP464006^56^.

The genome assembly data had been submitted to GenBank with accession number GCA_034766995.1^57^.

The genome annotation GFF, CDS sequences, and protein sequences are available in Figshare^58^.

## Technical Validation

### Assessment of the genome assembly and annotation

BUSCO was used to evaluate genome completeness at the chromosome-level according to the arthropoda_odb10 database. There are 1,003 (99.01%) complete genes, including 999 (98.62%) single-copy genes, 4 (0.39%) duplicated genes, 3 (0.30%) fragmented genes, and 7 (0.69%) missing genes identified (Table 5), indicating that the majority of conserved genes were assembled relatively complete and accurate. BUSCO was also used to evaluate the predicted gene set and about 970 (95.76%) (Table 5) of complete gene elements in the annotated gene set with 967 (95.46%) single-copy genes, 3 (0.30%) duplicated genes, 7 (0.69%) fragmented genes and 36 (3.55%) missing genes, indicating that the majority of conservative gene predictions were relatively complete and the prediction results are highly reliable.

**Table 5:** BUSCO evaluation statistics for genome assembly and annotation of *Acanthacorydalis orientalis*.

Type	Number	Percent (%)
Genome assembly		
Complete BUSCOs (C)	1,003	99.01
Complete and single-copy BUSCOs (S)	999	98.62
Complete and duplicated BUSCOs (D)	4	0.39
Fragmented BUSCOs (F)	3	0.3
Missing BUSCOs (M)	7	0.69
Total BUSCO groups searched	1,013	100
Gene annotation		
Complete BUSCOs (C)	970	95.76
Complete and single-copy BUSCOs (S)	967	95.46
Complete and duplicated BUSCOs (D)	3	0.3
Fragmented BUSCOs (F)	7	0.69
Missing BUSCOs (M)	36	3.55
Total BUSCO groups searched	1,013	100

### Supplementary information

Table 1

## Data Availability

No specific programs or codes were used in this study.
